# Comprehensive Genomic Profiling Across Diverse Tumors Using the OncoIndx® Assay: Real-World Insights From an Indian Cohort

**DOI:** 10.7759/cureus.115381

**Published:** 2026-08-28

**Authors:** Pramod K Julka, Deepika Arya, Afnan Bhasir, Trinica George, Ganesh Divekar, Gowhar Shafi

**Affiliations:** 1 Max Institute of Cancer Care, Max Smart Super Speciality Hospital, Saket, New Delhi, IND; 2 Clinical Development, 1Cell.Ai (OneCell Diagnostics India Pvt. Ltd.), Mumbai, IND; 3 Medical Genetics, 1Cell.Ai (OneCell Diagnostics India Pvt. Ltd.), Mumbai, IND

**Keywords:** actionable genomic alterations, comprehensive genomic profiling, liquid biopsy, next-generation sequencing (ngs), oncoindx®, pan-cancer profiling, precision oncology, somatic mutations

## Abstract

Background

Comprehensive genomic profiling (CGP) enables the simultaneous detection of actionable genomic alterations and predictive biomarkers across diverse malignancies. However, real-world molecular oncology data from Indian populations remain limited. This study aimed to characterize the genomic landscape of diverse solid tumors using the OncoIndx® (OneCell Diagnostics India Pvt. Ltd., Mumbai, India) CGP platform within routine Indian clinical practice.

Methods

This retrospective single-center study included patients with histologically confirmed malignancies who underwent CGP testing between June 2022 and July 2025. Genomic profiling was performed using the OncoIndx® 1080-gene next-generation sequencing (NGS) platform, with analyses focused on mutation frequency, alteration type, pathway enrichment, clinical-actionability categorization, and exploratory biomarkers including microsatellite instability (MSI), tumor mutational burden (TMB), homologous recombination deficiency (HRD), programmed death-ligand 1 (PD-L1) expression, and circulating tumor DNA (ctDNA) tumor fraction.

Results

The cohort comprised 39 samples representing diverse solid tumors, predominantly gynecological, gastrointestinal, breast, central nervous system (CNS), and thoracic malignancies. A total of 124 genomic alterations involving 69 unique genes were identified in 33 patients, while six patients demonstrated no detectable genomic alterations. TP53 was the most frequently altered gene (43.6%), followed by PIK3CA (17.9%), ATM (10.3%), and KRAS (10.3%). Category-wise analysis demonstrated substantial clinical relevance, with Categories 1 and 2 collectively accounting for 47.6% of all detected alterations. Pathway enrichment analysis revealed predominant involvement of TP53/cell-cycle (24.2%), PI3K/AKT/mTOR (15.3%), DNA damage repair/HRD-homologous recombination repair (HRD-HRR) (12.9%), and RTK/RAS/MAPK signaling pathways (9.7%). Somatic mutation mapping demonstrated marked inter-patient molecular heterogeneity, encompassing missense mutations, amplifications, frameshift variants, splice-site alterations, and fusion events. Exploratory biomarker analysis revealed predominantly low MSI and low TMB, although isolated MSI-high, elevated TMB, and HRD-positive tumors were identified. Variable concordance between tissue biopsy- and liquid biopsy-based PD-L1 assessment further underscored underlying tumor heterogeneity.

Conclusions

This real-world Indian cohort demonstrated extensive genomic heterogeneity across diverse solid tumors, with recurrent alterations involving TP53/cell-cycle, PI3K/AKT/mTOR, DNA damage repair, and RTK/RAS/MAPK pathways. OncoIndx®-based CGP enabled the broad molecular characterization and identification of clinically relevant genomic alterations and exploratory biomarkers. These findings provide descriptive real-world molecular insights from an underrepresented Indian population and support further prospective evaluation of the clinical utility and outcome implications of CGP in Indian oncology practice.

## Introduction

Precision oncology has transformed contemporary cancer therapeutics by shifting treatment paradigms from conventional histology-driven approaches toward molecularly guided individualized interventions [1]. Rapid advances in genomic technologies have revealed the extensive molecular heterogeneity underlying tumor biology, therapeutic responsiveness, resistance evolution, and clinical outcomes across malignancies [2]. Although genomically guided therapies currently benefit only a subset of patients, they have demonstrated superior and more durable clinical outcomes compared with conventional cytotoxic strategies [3]. Nevertheless, broader implementation of precision oncology remains constrained by limited access to advanced molecular diagnostics, economic barriers, infrastructure limitations, and the persistent complexity of genomic interpretation, particularly within resource-constrained healthcare systems.

Next-generation sequencing (NGS) has emerged as a central pillar of modern precision oncology by enabling the simultaneous high-throughput interrogation of multiple cancer-associated genes and clinically relevant biomarkers [4]. Unlike conventional single-gene assays, comprehensive genomic profiling (CGP) platforms facilitate the integrated characterization of diverse genomic alterations, including single nucleotide variants (SNVs), insertions/deletions (INDELs), copy number alterations (CNAs), splice-site variants, and gene rearrangements within a single assay [4,5]. Beyond the detection of actionable genomic alterations, broad-panel sequencing also enables the evaluation of clinically relevant biomarkers such as microsatellite instability (MSI), tumor mutational burden (TMB), and homologous recombination deficiency (HRD), thereby providing deeper insights into oncogenic pathway dysregulation, genomic instability, and potential responsiveness to targeted or immunotherapeutic strategies [6-8]. Among these, FoundationOne® CDx (F1CDx) (Foundation Medicine, Inc., Boston, MA, USA) became the first FDA-approved CGP assay for solid tumors in 2017 [9]. The assay interrogates 324 cancer-associated genes while concurrently assessing MSI and TMB, thereby enabling the integrated molecular characterization of solid tumors [9,10]. Increasingly, broad genomic profiling approaches have demonstrated the ability to identify clinically actionable alterations that may remain undetected using conventional single-gene or site-specific testing strategies. However, a considerable proportion of detected alterations continue to be classified as variants of uncertain significance (VUS), underscoring the persistent interpretative challenges within precision oncology [11].

The expanding clinical relevance of CGP has accelerated the development of multiple broad-panel sequencing platforms capable of integrated tissue biopsy- and liquid biopsy-based molecular profiling. In parallel, liquid biopsy approaches incorporating circulating tumor DNA (ctDNA) and circulating tumor cell (CTC)-based analyses have emerged as valuable complements to tissue-based profiling by enabling the minimally invasive longitudinal assessment of tumor evolution, clonal heterogeneity, and spatial genomic diversity [12,13]. Compared with conventional hotspot-focused assays, newer ultra-deep hybridization capture-based pan-cancer platforms such as OncoIndx® (OneCell Diagnostics India Pvt. Ltd., Mumbai, India) facilitate broader molecular interrogation across 1080 cancer-associated genes, including extensive exonic and intronic regions, fusion targets, and complex genomic biomarkers, while simultaneously supporting the detection of low-frequency subclonal alterations and dynamic resistance-associated events [14].

Despite substantial global advances in precision oncology, comprehensive real-world genomic datasets from Indian patients remain comparatively limited. Consequently, Indian and broader South Asian populations continue to be underrepresented within global molecular oncology databases, potentially restricting population-specific genomic interpretation and translational applicability. Accordingly, the primary aim of the present study was to characterize the genomic landscape of diverse solid tumors using the OncoIndx® CGP platform within routine clinical practice, with particular emphasis on recurrent genomic alterations and their clinical relevance. Secondary exploratory objectives were to examine the broader biological context of these alterations through pathway-level and somatic mutational analyses and to explore complementary molecular biomarkers that may further inform tumor characterization and biomarker-driven therapeutic stratification.

## Materials and methods

Study design and patient population

This retrospective, single-center cohort study was conducted at Max Institute of Cancer Care, Max Smart Super Speciality Hospital, Saket, a tertiary oncology center in New Delhi, India, to characterize the genomic landscape of diverse malignancies using CGP. Patients with histologically confirmed cancers who underwent genomic profiling using the OncoIndx® 1080-gene NGS platform as part of routine clinical care between June 2022 and July 2025 were included. Clinical and molecular data were retrospectively extracted from anonymized medical records and genomic profiling reports to evaluate mutational profiles, clinically actionable alterations, and pathway-level genomic patterns. Tumors were categorized according to both histological subtype and broader organ system-based classifications. The study did not evaluate the determinants, accessibility, or overall utilization of CGP among the broader eligible patient population. It was conducted in accordance with the principles of the Declaration of Helsinki and adhered to Good Clinical Practice (GCP) guidelines. Ethical approval was granted by the Max Healthcare Ethics Committee (approval number: BHR/RS/MSSSH/GMHRCMS/MHEC/ONCO/26-02; date: June 19, 2026).

Study objectives and endpoints

The primary objective was to characterize the genomic landscape of diverse solid tumors using the OncoIndx® CGP platform. The primary endpoints included the prevalence of detectable genomic alterations, frequency of recurrently altered genes, number and types of genomic alterations, and their categorization according to clinical actionability and interpretative relevance. Secondary exploratory endpoints included the distribution of genomic alterations across major oncogenic pathways, somatic mutational patterns, and VUS. Additional exploratory biomarker endpoints included MSI, TMB, HRD and related genomic instability metrics, programmed death-ligand 1 (PD-L1) expression, and ctDNA tumor fraction, where available.

NGS methodology and genomic analysis

CGP was performed at OneCell Diagnostics India Pvt. Ltd. using the OncoIndx® platform, a targeted NGS assay comprising a 1080-gene panel designed for broad molecular characterization of solid tumors. The assay incorporated both tissue biopsy-based and liquid biopsy-based profiling approaches. Tumor-derived ctDNA and formalin-fixed paraffin-embedded (FFPE)-derived DNA were extracted using commercially available extraction kits (QIAamp MinElute ccfDNA Kit, QIAGEN, Hilden, Germany). Extracted nucleic acids underwent quality assessment using the TapeStation 4200 system (Agilent Technologies, Santa Clara, CA, USA), and samples demonstrating ≥80% ccfDNA content were considered eligible for downstream analysis. DNA concentration was quantified using the Qubit 4.0 fluorometer (Thermo Fisher Scientific, Waltham, MA, USA). Following quality control, Illumina-compatible sequencing libraries were prepared using a hybridization capture-based target enrichment methodology and sequenced on the NextSeq 2000 platform (Illumina, San Diego, CA, USA) employing paired-end sequencing chemistry (2 × 150 bp). Raw sequencing data underwent FASTQ-level and overall sequencing quality assessment, including evaluation of Q30, on-target coverage, and sequencing depth. Average sequencing depths were approximately 2,000× for tissue and 10,000× for blood/liquid biopsy specimens. Following adapter and barcode trimming, sequencing reads were processed using the proprietary iCare™ bioinformatics platform (Version 2, 2025; OneCell Diagnostics India Pvt. Ltd., Mumbai, India) and aligned to the GRCh38 human reference genome. Variant filtering required a variant quality score of >15, ≥5 supporting reads, and a variant allele frequency (VAF) of ≥0.1%; variants exhibiting high strand bias were excluded. CNAs were identified using a read-depth-ratio-based algorithm optimized for hybrid-capture targeted sequencing, incorporating the correction for tumor purity, ploidy, low-coverage regions, and appropriate normal-pool comparisons. Variants were annotated and prioritized using ClinVar and the curated iCare™ knowledge base, supplemented by in silico prediction tools where applicable. Additional analytical parameters are summarized in Table 1, while the analytical characteristics and technical validation of the OncoIndx® assay have been described previously [14].

**Table 1:** Sequencing and bioinformatics parameters for OncoIndx® CGP CGP: comprehensive genomic profiling; QC: quality control; VAF: variant allele frequency; CNV: copy number variation; SIFT: Sorting Intolerant From Tolerant; PolyPhen: Polymorphism Phenotyping

Analytical parameter	Specification
CGP platform	OncoIndx® 1080-gene panel
Target enrichment	Hybridization capture-based
Sequencing platform	Illumina NextSeq 2000
Sequencing chemistry	Paired-end, 2 × 150 bp
Mean sequencing depth (tissue)	~2,000×
Mean sequencing depth (blood/liquid biopsy)	~10,000×
Reference genome	GRCh38
Bioinformatics platform	iCare™, Version 2 (2025)
Post-sequencing QC	FASTQ QC, Q30, on-target coverage, sequencing depth
Minimum total read depth	≥30×
Variant quality threshold	>15
Minimum supporting reads	≥5
Minimum VAF	≥0.1%
Strand-bias filtering	High strand-bias variants excluded
CNV detection	Read-depth-ratio-based copy number calling optimized for hybrid-capture targeted sequencing
CNV analysis	Segment-level copy number estimation, with tumor purity/ploidy and low-coverage correction and normal-pool comparison, as applicable
Variant annotation/prioritization	ClinVar and the curated iCare™ knowledge base, supplemented by in silico prediction tools including SIFT and PolyPhen, where applicable
Missing biomarker data	Recorded as missing/not available; no imputation; not classified as negative

Exploratory biomarkers, including MSI, TMB, HRD-related metrics, PD-L1 expression, and ctDNA tumor fraction, were analyzed where available in the source genomic reports. Unavailable or missing biomarker assessments were recorded as missing/not available, were not imputed, and were not classified as negative; biomarker-specific summaries were therefore based on available assessments.

Genomic alterations were analyzed for mutation frequency, recurrently altered genes, alteration type, and patient-level mutational patterns. Gene-level frequencies were calculated on a per-patient/sample basis, with each gene counted only once per sample irrespective of multiple alterations or repeat entries. The assay-level clinical interpretation of detected variants was based on established evidence-based annotation, whereas, for the purposes of the present retrospective analysis, genomic alterations were subsequently grouped into seven descriptive clinical-actionability categories according to their reported therapeutic, predictive, prognostic, biological, or interpretative relevance. Category 1 represented alterations with established therapeutic relevance/high clinical actionability; Category 2, alterations with guideline-supported or strong published clinical evidence; Category 3, alterations with emerging or potential clinical actionability; Category 4, alterations with predominantly biological, prognostic, or predictive relevance without established therapeutic actionability; Category 5, variants of uncertain clinical significance; Category 6, alterations with limited or low-level evidence or context-dependent relevance; and Category 7, alterations for which no currently interpretable clinical actionability was identified. This seven-category framework was used as a descriptive analytical stratification for the present study and should not be interpreted as equivalent to the formal Association for Molecular Pathology/American Society of Clinical Oncology/College of American Pathologists (AMP/ASCO/CAP) somatic variant classification system. The AMP/ASCO/CAP consensus framework classifies somatic variants into four tiers according to clinical significance: Tier I (strong clinical significance), Tier II (potential clinical significance), Tier III (unknown clinical significance), and Tier IV (benign or likely benign) [11]. Further, variants were grouped into major oncogenic pathways, including TP53/cell-cycle, PI3K/AKT/mTOR, DNA damage repair/homologous recombination repair (HRR), and RTK/RAS/MAPK signaling. Exploratory biomarkers, including MSI, TMB, HRD-related metrics, PD-L1 expression, and ctDNA tumor fraction, were assessed where available. Somatic mutation mapping and oncoplot-style visualizations were generated to depict the distribution of genomic alterations across the cohort.

Sample size calculation

As this was a retrospective, exploratory study, no formal sample size calculation was performed. The sample size was determined by the number of eligible patients available during the study period. The study was intended to provide a descriptive characterization of the genomic landscape and actionable alterations in a real-world Indian cohort.

Statistical analysis

Descriptive statistics were used to summarize demographic, clinicopathological, and molecular characteristics. Continuous variables were presented as mean ± standard deviation (SD) or median with range, while categorical variables were summarized as frequencies and percentages. Genomic alteration frequencies, category distributions, pathway enrichment, and biomarker prevalence were analyzed descriptively. Descriptive analyses were performed using Microsoft Excel 2021 (Microsoft Corp., Redmond, WA, USA). Somatic mutation mapping and biomarker heatmap visualizations were generated using the R statistical software (Version 4.3.2; R Foundation for Statistical Computing, Vienna, Austria).

## Results

The study cohort comprised 39 samples, including 24 (61.5%) female and 15 (38.5%) male patients. The mean age of the study cohort was 55.8 ± 13.2 years. System-level classification demonstrated a predominance of gynecological malignancies (n = 12), followed by gastrointestinal cancers (n = 6), breast cancer (n = 5), central nervous system (CNS) tumors (n = 4), thoracic malignancies (n = 4), and other tumor types (n = 8). At the individual cancer-type level, ovarian cancer, breast cancer, uterine cancer, brain tumors, colorectal cancer, and lung cancer constituted the major diagnostic groups. Table 2 summarizes the demographic distribution, tumor classification, and overall genomic landscape of the analyzed cohort.

**Table 2:** Study population composition and genomic landscape across the analyzed cohort CNS: central nervous system

Variables	n = 39
Gender, n (%)	
Female	24 (61.5)
Male	15 (38.5)
System-level group, n (%)	
Gynecologic malignancy	12 (30.8)
Gastrointestinal cancer	6 (15.4)
Breast cancer	5 (12.8)
CNS cancer	4 (10.3)
Thoracic malignancy	4 (10.3)
Others	8 (20.5)
Genomic alteration, n (%)	
Present	33 (84.6)
Absent	6 (15.4)
Number of alterations	124
Number of alterations per patient, mean ± SD	3.2 ± 2.59
Frequently altered genes, n (%)	
TP53	17 (43.6)
PIK3CA	7 (17.9)
ATM	4 (10.3)
KRAS	4 (10.3)
CDKN2A	3 (7.7)
ARID1A	2 (5.1)
BRCA1	2 (5.1)
BRCA2	2 (5.1)

Across the cohort, a total of 124 genomic alterations involving 69 unique genes were identified in 33 patients, while six patients demonstrated no detectable genomic alterations. The most frequently altered genes included TP53 (17/39, 43.6%), followed by PIK3CA (7/39, 17.9%), ATM (4/39, 10.3%), and KRAS (4/39, 10.3%). Less frequent alterations were observed in CDKN2A (3/39, 7.7%), ARID1A (2/39, 5.1%), BRCA1 (2/39, 5.1%), and BRCA2 (2/39, 5.1%). TP53 emerged as the predominant recurrently altered gene, with a substantially higher frequency relative to other genes, followed by PIK3CA. The mean number of alterations per patient was 3.2 ± 2.59, reflecting marked inter-patient molecular heterogeneity. Recurrent within-patient alterations were particularly observed in TP53, PIK3CA, ARID1A, and CDKN2A.

Clinical-actionability categorization demonstrated a broad distribution of genomic alterations across the seven predefined descriptive categories (Table 3).

**Table 3:** Clinical-actionability categorization of genomic alterations ^Categories represent a study-specific descriptive stratification of clinical actionability and interpretative relevance and are not equivalent to the AMP/ASCO/CAP Tier I-IV somatic variant classification system. AMP: Association for Molecular Pathology; ASCO: American Society of Clinical Oncology; CAP: College of American Pathologists

Clinical-actionability categories^	Alteration, n (%)	Patient count, n (%)
Category 1: Established therapeutic relevance/high clinical actionability	18 (14.5)	13 (33.3)
Category 2: Guideline-supported or strong published clinical evidence	41 (33.1)	18 (46.2)
Category 3: Emerging/potential clinical actionability	9 (7.3)	9 (23.1)
Category 4: Biological, prognostic, or predictive relevance	14 (11.3)	11 (28.2)
Category 5: Uncertain or limited clinical significance	9 (7.3)	3 (7.7)
Category 6: Limited/low-level evidence or context-dependent relevance	10 (8.1)	6 (15.4)
Category 7: No currently interpretable clinical actionability	23 (18.5)	18 (46.2)

Category 1 comprised 18/124 (14.5%) alterations and was represented in 13 patients (33.3%), while Category 2 constituted the largest group, comprising 41/124 (33.1%) alterations and occurring in 18 patients (46.2%). Categories 3 and 4 accounted for 9/124 (7.3%) and 14/124 (11.3%) alterations, respectively. Variants of uncertain or limited clinical significance classified as Category 5 accounted for 9/124 (7.3%) alterations, whereas Categories 6 and 7 comprised 10/124 (8.1%) and 23/124 (18.5%) alterations, respectively. Patient-level proportions across clinical-actionability categories are not mutually exclusive, as individual patients may harbor multiple genomic alterations assigned to different categories. Overall, the distribution demonstrated considerable heterogeneity in the level of clinical and biological interpretability of the genomic alterations identified across the cohort.

Pathway-level analysis demonstrated that genomic alterations were predominantly enriched within key oncogenic and tumor suppressor pathways (Table 4).

**Table 4:** Pathway-level annotations HRD: homologous recombination deficiency; HRR: homologous recombination repair; WNT: Wingless-related integration site; MSI: microsatellite instability

Key oncogenic pathway	N (%)
TP53/cell cycle	30 (24.2)
PI3K/AKT/mTOR	19 (15.3)
DNA damage repair/HRD-HRR	16 (12.9)
RTK/RAS/MAPK	12 (9.7)
Chromatin remodeling/epigenetic	11 (8.9)
WNT/β-catenin	4 (3.2)
Hedgehog signaling	3 (2.4)
Mismatch repair/MSI	3 (2.4)
Fusion/transcriptional regulation	2 (1.6)
Apoptosis	1 (0.8)
Cell adhesion/Hippo	1 (0.8)
Cell signaling/phosphatase	1 (0.8)
Cytoskeleton/migration	1 (0.8)
Hormone receptor signaling	1 (0.8)
mTOR/metabolic signaling	1 (0.8)
RNA processing	1 (0.8)
RNA splicing	1 (0.8)
Other/unmapped	16 (12.9)

Alterations involving the TP53/cell-cycle pathway were the most common (30/124, 24.2%), followed by PI3K/AKT/mTOR signaling (19/124, 15.3%) and DNA damage repair/HRD-HRR pathways (16/124, 12.9%). Additional involvement was observed in the RTK/RAS/MAPK signaling axis (12/124, 9.7%) and chromatin remodeling/epigenetic pathways (11/124, 8.9%). Less frequent alterations affected the Wingless-related integration site (WNT)/β-catenin pathway (3.2%), Hedgehog signaling (2.4%), and mismatch repair/MSI pathways (2.4%), while fusion/transcriptional regulatory alterations accounted for 1.6% of cases. Other pathways, including apoptosis, cell adhesion/Hippo signaling, cytoskeletal regulation, hormone receptor signaling, metabolic/mTOR signaling, and RNA processing/splicing, each contributed marginally (0.8% each). Notably, 12.9% of alterations were categorized as other/unmapped, representing variants that could not be assigned to established canonical pathways. Collectively, the pathway distribution highlighted a biologically coherent landscape dominated by cell-cycle regulation, survival signaling, and DNA repair mechanisms.

Somatic mutation mapping further demonstrated a broad spectrum of alteration types across the cohort, including missense mutations, amplifications, nonsense mutations, frameshift alterations, splice-site variants, and fusion/rearrangement events (Figure 1).

**Figure 1:**
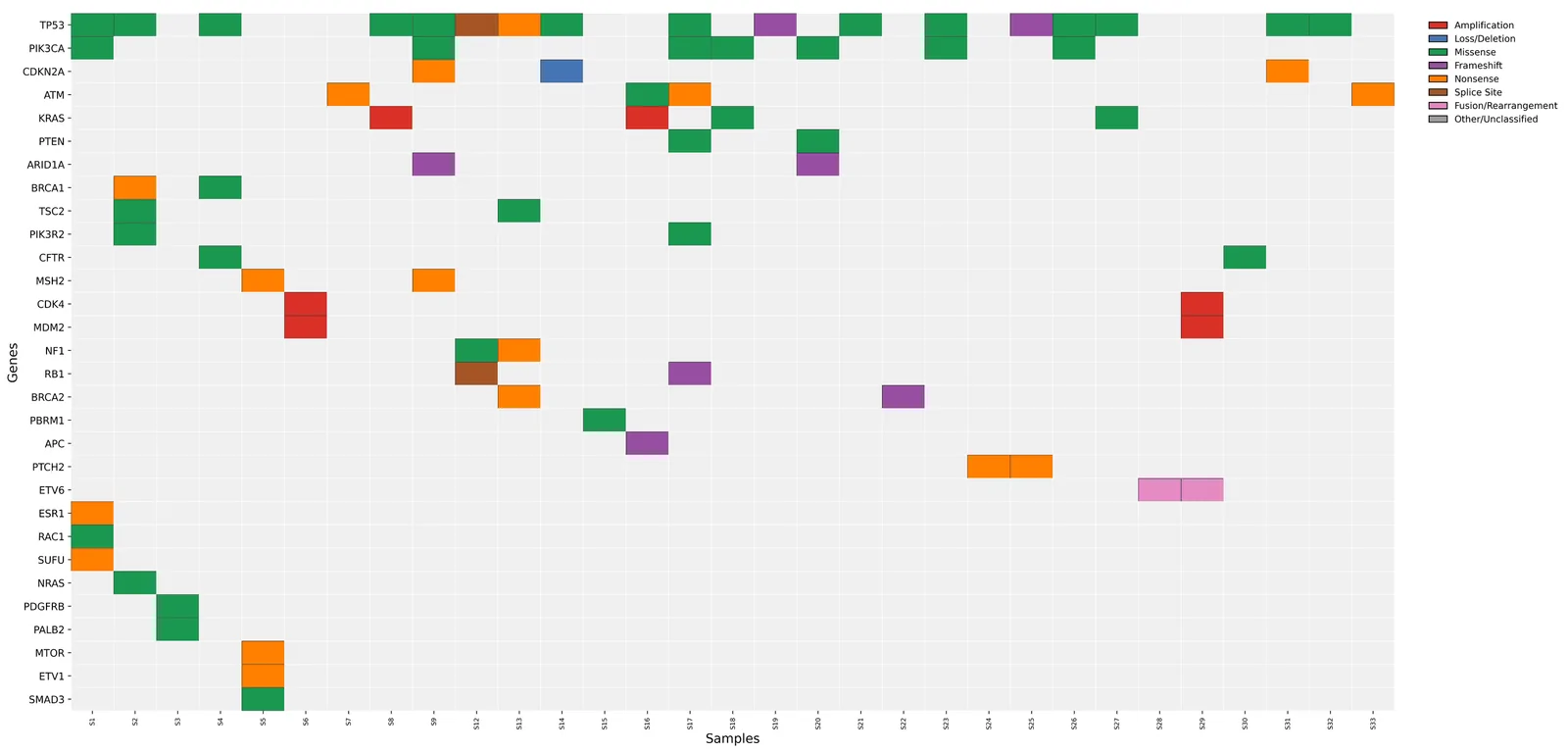
Somatic mutation map illustrating the distribution of genomic alterations across diverse solid tumors stratified by key oncogenic pathways

Missense variants represented the predominant alteration category. Recurrent amplifications were identified in oncogenic drivers such as KRAS, CDK4, and MDM2, whereas truncating and frameshift alterations involving tumor suppressor genes including TP53, RB1, BRCA2, and APC suggested potential loss-of-function events. Fusion/rearrangement alterations, including ETV6 rearrangements, were identified in a subset of patients. Overall, the somatic mutation map highlighted substantial inter-patient genomic diversity with concurrent alterations affecting multiple biologically and therapeutically relevant oncogenic pathways, underscoring the molecular complexity of the cohort.

A diverse spectrum of VUS was identified across the cohort, involving multiple biologically relevant pathways associated with DNA damage repair, epigenetic regulation, receptor tyrosine kinase signaling, and cell-cycle control (Table 3). Recurrent alterations in genes implicated in chromatin remodeling and epigenetic regulation included KMT2C, SETD2, SETDB1, and DNMT3A, with several variants demonstrating subclonal architecture, including SETDB1 p.E657 and KMT2C p.R886H (VAF ~3%). Alterations involving DNA repair pathway genes such as FANCA, MUTYH, and ERCC5 were also observed, suggesting possible underlying genomic instability in a subset of samples. VUS affecting oncogenic signaling pathways included alterations in ERBB2 (p.R456C; VAF 17%), FGFR4 (including p.G388R and p.L698Q), AKT3, MTOR, and KIT, indicating potential perturbations in RTK/PI3K/AKT/mTOR signaling axes. Additional variants were detected in transcriptional regulators and signaling mediators such as RELA, MED12, RIT1, and RXRA. Several samples demonstrated high-VAF VUS, including alterations in BRCA2 (VAF 63%), PTK2 (VAF 71%), LRP5 (VAF 52%), and KRT222/CDKN1B (VAF 40-47% in plasma DNA), suggesting potential clonal or dominant genomic events despite their current uncertain classification. Hormone signaling-related alterations, including ESR1 mutations (p.L536P and p.H524R), were also identified at lower VAFs (6-7%), which may have emerging implications in endocrine resistance mechanisms. A subset of samples demonstrated no detectable VUS, further emphasizing inter-sample heterogeneity in mutational burden and variant classification. Overall, the VUS landscape highlighted substantial genomic diversity involving multiple cancer-relevant pathways, with several biologically plausible variants warranting further functional and clinical investigation.

Exploratory biomarker assessment included MSI, TMB, HRD, loss of heterozygosity (LOH), telomeric allelic imbalance (TAI), large-scale state transitions (LST), PD-L1 expression, and ctDNA tumor fraction (Figure 2).

**Figure 2:**
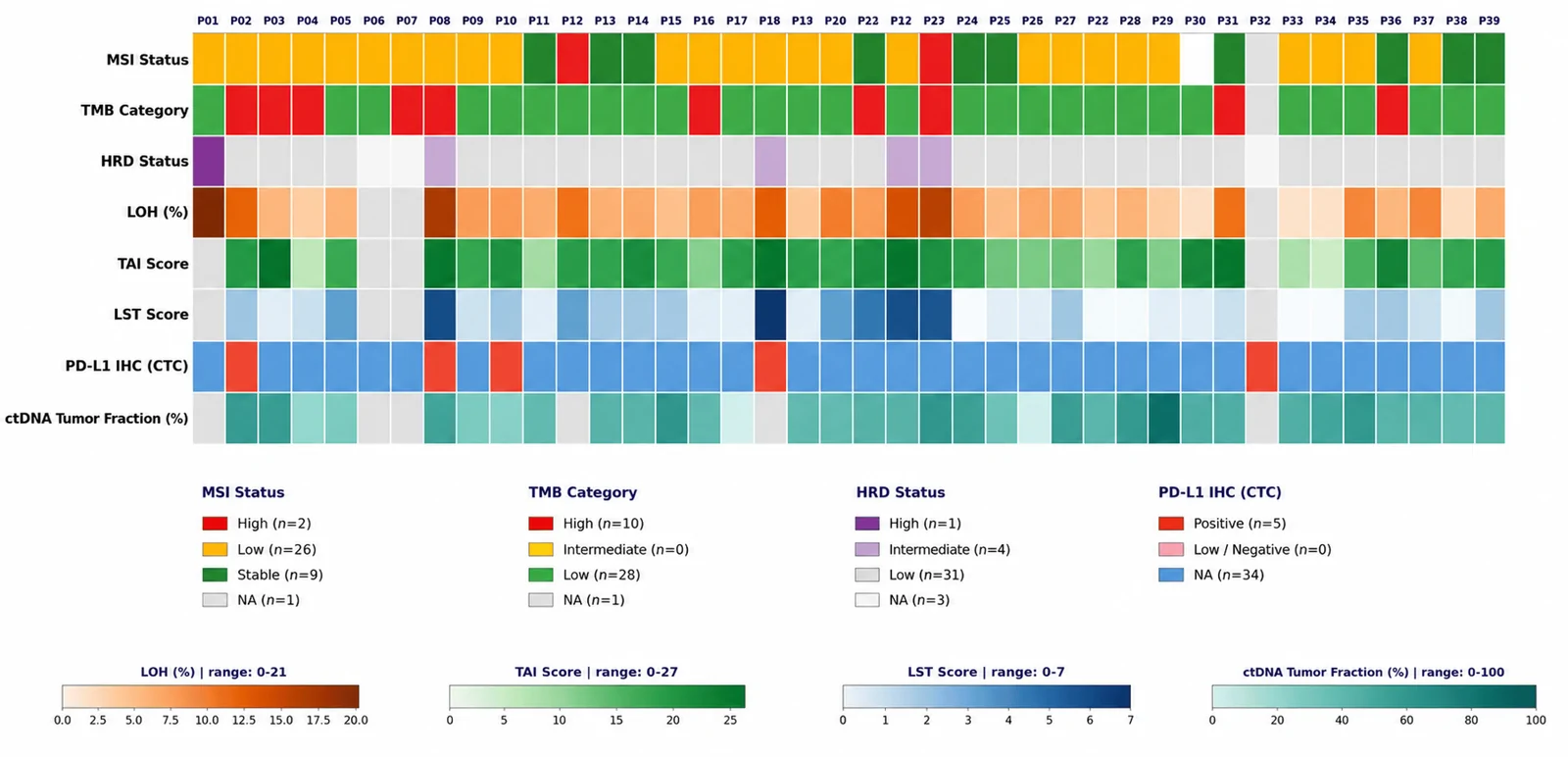
Exploratory biomarker landscape across the study cohort (n = 39) MSI: microsatellite instability; TMB: tumor mutational burden; HRD: homologous recombination deficiency; LOH: loss of heterozygosity; TAI: telomeric allelic imbalance; LST: large-scale state transitions; PD-L1: programmed death-ligand 1; ctDNA: circulating tumor DNA; IHC: immunohistochemistry; CTC: circulating tumor cell

The comprehensive exploratory biomarker profile of the study cohort is illustrated in Figure 2. MSI status was predominantly low, with MSI-high identified in only two samples and nine cases classified as MSI-stable. TMB was generally low across the cohort, although elevated TMB was observed in 10 samples. HRD scores were largely low, with only one sample demonstrating high HRD. Corresponding variability was also observed in genomic instability metrics including LOH, TAI, and LST, with high LOH identified in one sample. Among five patients identified as PD-L1-positive on CTC-based analysis, concordance with tissue-based immunohistochemistry (IHC) was observed in only one case, whereas two cases demonstrated discordance (CTC-positive/IHC-negative), and IHC data were unavailable in two cases. This limited concordance suggests that although agreement between tissue biopsy- and liquid biopsy-based PD-L1 assessment may occur in a subset of patients, CTC-based evaluation may offer enhanced sensitivity in capturing dynamic and spatial tumor heterogeneity, thereby serving as a complementary and, in selected contexts, potentially superior approach to single-site tissue assessment. The ctDNA tumor fraction demonstrated marked variability, ranging from low levels to as high as 100%, reflecting substantial heterogeneity in tumor burden and ctDNA shedding dynamics. Collectively, the biomarker landscape demonstrated predominantly low MSI and TMB, variable genomic instability indices, and heterogeneous liquid biopsy profiles, underscoring the biological complexity and potential relevance of biomarker-driven stratification within the cohort.

## Discussion

The present study provides comprehensive real-world insights into the molecular landscape of diverse solid tumors within an Indian precision oncology setting using the OncoIndx® CGP platform. The findings demonstrate extensive inter-patient molecular heterogeneity characterized by the recurrent dysregulation of TP53/cell-cycle signaling, PI3K/AKT/mTOR pathways, DNA damage repair mechanisms, and RTK/RAS/MAPK signaling cascades. Importantly, a substantial proportion of patients harbored clinically actionable or potentially actionable genomic alterations, underscoring the growing clinical relevance of integrated broad-panel genomic profiling within routine oncology practice, particularly within resource-constrained healthcare systems [15,16].

The genomic distribution observed in the present cohort demonstrated broad concordance with previously published Indian and global pan-cancer genomic profiling datasets [17-19]. Similar to prior Indian real-world CGP studies utilizing F1CDx testing, recurrent enrichment of TP53 alterations together with PI3K-pathway dysregulation emerged as dominant biological themes across multiple tumor types [17]. Such concordance across distinct sequencing platforms and heterogeneous tumor populations supports the reproducibility of major oncogenic programs underlying tumor progression in Indian cancer cohorts. The predominance of TP53 alterations observed in the current study is biologically significant because TP53 dysfunction is strongly associated with genomic instability, clonal evolution, therapeutic resistance, and adverse oncologic outcomes across malignancies [18,19]. In parallel, recurrent alterations involving PIK3CA, KRAS, ATM, BRCA1/2, CDKN2A, and ARID1A further highlighted the biological importance of proliferative signaling, DNA repair dysfunction, chromatin remodeling, and cell-cycle dysregulation within the analyzed cohort. The pathway-level enrichment of the PI3K/AKT/mTOR axis observed in the present analysis closely parallels findings from previously published breast and gynecologic cancer genomic profiling studies, where recurrent PIK3CA, PTEN, and PI3K-pathway alterations represented major therapeutically targetable molecular events [17,20,21]. In the Indian F1CDx cohort, breast and gynecologic tumors similarly demonstrated prominent PI3K-pathway activation accompanied by recurrent TP53 disruption and amplification-driven oncogenic programs [17]. These observations are clinically important because PI3K-pathway alterations increasingly influence therapeutic decision-making through the incorporation of PI3K inhibitors, AKT inhibitors, mTOR inhibitors, and combinatorial endocrine-targeted approaches in contemporary oncology practice [17,22].

An important observation in the present analysis was the substantial proportion of genomic alterations assigned to the higher clinical-actionability categories. Categories 1 and 2 collectively accounted for 47.6% of the detected alterations. However, as treatment selection, molecularly matched therapy, treatment response, and clinical outcomes were not evaluated, these findings demonstrate potential molecular actionability rather than established clinical utility or therapeutic benefit. Similar findings were reported in the Indian F1CDx experience, where same-tumor FDA-approved therapeutic recommendations were identified in 41.7% of patients and tumor-agnostic or off-label recommendations in approximately 50% [17]. Collectively, these observations reinforce prior evidence demonstrating that broad-panel NGS substantially increases the likelihood of identifying clinically actionable alterations compared with conventional single-gene or site-specific testing strategies [15,23]. However, the study also highlights the persistent distinction between biological actionability and real-world therapeutic accessibility. Although multiple alterations theoretically qualify for targeted interventions, practical implementation within low- and middle-income healthcare systems remains constrained by treatment availability, economic burden, reimbursement limitations, and restricted access to molecularly matched therapies [17].

The present study additionally demonstrated extensive inter-patient molecular heterogeneity, reflected by substantial variability in alteration burden, mutation type, pathway involvement, biomarker expression, and within-patient co-alteration patterns. Similar genomic complexity has been consistently reported across large-scale pan-cancer sequencing studies and real-world NGS datasets [17,24]. Missense variants represented the predominant alteration category in our cohort; however, amplifications, splice-site variants, frameshift alterations, rearrangements, and fusion events were also observed across biologically significant oncogenic drivers. Recurrent amplifications involving KRAS, MDM2, and CDK4, together with truncating events affecting TP53, APC, RB1, and BRCA2, highlighted the coexistence of oncogenic activation and tumor suppressor inactivation contributing to tumor progression and resistance evolution. Such multidimensional co-alteration patterns further underscore the limitations of narrow hotspot testing strategies, which may inadequately capture the broader molecular architecture increasingly recognized in advanced malignancies.

The pathway-level distribution observed in this study demonstrated a biologically coherent genomic landscape predominantly characterized by the dysregulation of cell-cycle control, proliferative signaling, and DNA repair mechanisms. TP53/cell-cycle pathway alterations were the most frequent, consistent with the established role of TP53 dysfunction in genomic instability, tumor progression, and therapeutic resistance across multiple malignancies [25]. Frequent alterations involving the PI3K/AKT/mTOR and RTK/RAS/MAPK pathways further highlighted the importance of oncogenic survival signaling and proliferative cascades in tumor development and progression [26]. Additionally, the substantial representation of DNA damage repair/HRD-HRR pathway alterations emphasized the growing clinical relevance of genomic instability biomarkers and their therapeutic implications, particularly in the context of poly(ADP-ribose) polymerase (PARP) inhibitor-based precision oncology strategies [27]. Collectively, these findings reinforce the biological validity of the detected genomic alterations while underscoring the utility of broad-panel CGP in identifying clinically relevant molecular vulnerabilities across diverse solid tumors.

An additional major observation in the present study was the broad spectrum of VUS identified across multiple biologically plausible oncogenic pathways. Despite increasing standardization, interpretation of novel, low-frequency, or population-specific variants remains a major challenge within precision oncology [11]. Multiple VUS identified in our cohort involved chromatin remodeling genes (KMT2C, SETD2, SETDB1), DNA repair genes (FANCA, MUTYH, ERCC5), and RTK/PI3K signaling mediators (ERBB2, FGFR4, MTOR, AKT3). Importantly, several VUS demonstrated high variant allele frequencies suggestive of potential clonal significance despite incomplete functional characterization. Similar concerns regarding the interpretation of VUS and underrepresentation of Asian genomic datasets have been highlighted in prior NGS studies [11,17]. These observations collectively emphasize the urgent need for expansion of population-specific genomic databases, functional validation studies, and longitudinal clinico-genomic correlation efforts within Indian and South Asian populations.

The integration of both tissue biopsy-based and liquid biopsy-based profiling constituted a major methodological strength of the present study [14]. Liquid biopsy approaches incorporating ctDNA and CTC-based analyses increasingly serve as valuable complementary tools to tissue-based profiling because they facilitate minimally invasive longitudinal monitoring, dynamic assessment of resistance evolution, and improved capture of spatial and temporal tumor heterogeneity [12,28]. The marked variability observed in ctDNA tumor fraction likely reflects differences in tumor burden, vascularity, metastatic dissemination, and ctDNA shedding dynamics. Furthermore, the observed discordance between tissue-based and CTC-based PD-L1 assessment supports emerging evidence suggesting that liquid biopsy-based biomarker evaluation may more effectively capture dynamic tumor heterogeneity compared with single-site tissue sampling [28]. In a subset of patients, no detectable genomic alterations were identified despite adequate assay performance. The absence of identifiable variants in these cases may reflect a combination of biological and technical factors, including low tumor purity, reduced ctDNA shedding, and ultra-low variant allele frequencies below the analytical limit of detection of the OncoIndx® assay (VAF 0.1%) [29]. Additionally, intratumoral heterogeneity and spatially restricted subclonal alterations may further limit mutation capture within a single analyzed specimen [30]. These findings underscore the inherent biological complexity and technical limitations associated with CGP assays.

Exploratory biomarker analysis further demonstrated predominantly low MSI and low-to-intermediate TMB across the cohort, findings that parallel previously reported Indian real-world genomic datasets [17]. Nevertheless, isolated MSI-high, elevated TMB, and HRD-positive tumors identified within the cohort remain clinically important because they may identify candidates for tumor-agnostic immunotherapeutic or DNA repair-targeted treatment strategies. The identification of such molecular subsets reinforces the importance of comprehensive biomarker interrogation beyond conventional site-specific testing paradigms.

Despite substantial global advances in precision oncology, implementation of CGP within low- and middle-income countries remains challenging because of economic constraints, infrastructure limitations, restricted reimbursement pathways, inadequate genomic expertise, and limited therapeutic accessibility [15]. Real-world genomic datasets from Indian populations remain comparatively sparse, resulting in the underrepresentation of region-specific molecular signatures within global oncology databases. The present study therefore contributes descriptive real-world molecular data from Indian clinical practice while highlighting the need for larger multicentric genomic registries and prospective studies incorporating patterns of CGP utilization, accessibility, treatment selection, and longitudinal clinical outcomes.

Strengths and limitations

The principal strengths of the present study include the use of a broad 1080-gene CGP platform integrating tissue biopsy- and liquid biopsy-based profiling, evaluation of clinically relevant genomic alterations and exploratory biomarkers, and inclusion of diverse solid tumors reflective of real-world Indian oncology practice. Importantly, the study contributes molecular oncology data from an underrepresented Indian population. Several limitations should be considered when interpreting these findings. The retrospective, single-center design and inclusion of patients undergoing molecular profiling as part of routine care may have introduced selection bias. The relatively small and heterogeneous cohort limited statistical power for disease-specific analyses and tumor-specific interpretation. Furthermore, pooling tissue- and ctDNA-derived alterations may have introduced heterogeneity owing to differences in specimen characteristics, tumor DNA abundance, variant detectability, and biological representation. As no formal hypothesis testing was performed, the findings are descriptive, exploratory, and hypothesis-generating rather than confirmatory. The absence of an independent validation cohort further limits generalizability. No detectable genomic alterations were identified in 6/39 (15.4%) patients, which may reflect biological as well as technical factors, including tumor content, ctDNA shedding, sampling heterogeneity, and alterations below the analytical detection threshold. Clinical-actionability findings should also be interpreted cautiously, as alteration-level proportions do not directly represent patient-level actionability. Finally, the absence of longitudinal treatment-response and survival data precluded the assessment of the therapeutic or prognostic implications of the identified alterations. Larger multicentric studies incorporating specimen-specific analyses, independent validation, and longitudinal clinico-genomic outcomes are warranted.

## Conclusions

This real-world Indian study demonstrates substantial genomic heterogeneity across diverse solid tumors, characterized by recurrent alterations involving TP53/cell-cycle, PI3K/AKT/mTOR, DNA damage repair, and RTK/RAS/MAPK pathways. CGP using the OncoIndx® platform enabled the broad molecular characterization and identification of clinically relevant genomic alterations and exploratory biomarkers across multiple malignancies. However, in the absence of treatment-response and longitudinal outcome data, these findings should be interpreted as descriptive and hypothesis-generating rather than evidence of direct clinical benefit. Collectively, the study contributes real-world molecular data from an underrepresented Indian population and provides a basis for larger prospective studies evaluating the clinical utility, accessibility, and outcome implications of CGP in Indian oncology practice.
